# Correction: Discovery of penicillic acid as a chemical probe against tau aggregation in Alzheimer's disease

**DOI:** 10.1039/d6sc90134d

**Published:** 2026-06-23

**Authors:** Jennifer Shyong, Jinliang Wang, Quoc-Dung Tran Huynh, Marina Fayzullina, Bo Yuan, Ching-Kuo Lee, Thomas Minehan, Paul M. Seidler, Clay C. C. Wang

**Affiliations:** a Department of Pharmacology and Pharmaceutical Sciences, Alfred E. Mann School of Pharmacy and Pharmaceutical Sciences, University of Southern California Los Angeles California 90089 USA clayw@usc.edu pseidler@usc.edu; b Department of Chemistry, University of Southern California, Dornsife College of Letters, Arts, and Sciences Los Angeles California 90089 USA; c School of Pharmacy, College of Pharmacy, Taipei Medical University Taipei 11031 Taiwan; d PhD Program in Clinical Drug Development of Herbal Medicine, College of Pharmacy, Taipei Medical University Taipei 11031 Taiwan; e Department of Chemistry and Biochemistry, California State University, Northridge Northridge California 91330 USA

## Abstract

Correction for ‘Discovery of penicillic acid as a chemical probe against tau aggregation in Alzheimer's disease’ by Jennifer Shyong *et al.*, *Chem. Sci.*, 2024, **15**, 20467–20477, https://doi.org/10.1039/D4SC05469E.

The authors regret that funding details were incorrect in the Acknowledgements section of the original article. The corrected Acknowledgements section for this article is shown below.

**Acknowledgements**

We thank the donors and their families, without whom this work would not have been possible. We are grateful to Simulations Plus for providing a license for ADMET Predictor® through the University + program. We also thank the BrightFocus Foundation (A2023016S). TM acknowledges support from the National Science Foundation (CHE-2003261). Figures were created with https://www.biorender.com/.

The Royal Society of Chemistry apologises for these errors and any consequent inconvenience to authors and readers.

